# Natural Compounds and Biopolymers-Based Hydrogels Join Forces to Promote Wound Healing

**DOI:** 10.3390/pharmaceutics15010271

**Published:** 2023-01-12

**Authors:** Federica Falbo, Umile Gianfranco Spizzirri, Donatella Restuccia, Francesca Aiello

**Affiliations:** Department of Pharmacy, Health, and Nutritional Sciences, University of Calabria, Edificio Poli-Funzionale, 87036 Rende, CS, Italy

**Keywords:** wound healing, antibacterial activity, polyphenols, biopolymers

## Abstract

Rapid and complete wound healing is a clinical emergency, mainly in pathological conditions such as Type 2 Diabetes mellitus. Many therapeutic tools are not resolutive, and the research for a more efficient remedial remains a challenge. Wound dressings play an essential role in diabetic wound healing. In particular, biocompatible hydrogels represent the most attractive wound dressings due to their ability to retain moisture as well as ability to act as a barrier against bacteria. In the last years, different functionalized hydrogels have been proposed as wound dressing materials, showing encouraging outcomes with great benefits in the healing of the diabetic wounds. Specifically, because of their excellent biocompatibility and biodegradability, natural bioactive compounds, as well as biomacromolecules such as polysaccharides and protein, are usually employed in the biomedical field. In this review, readers can find the main discoveries regarding the employment of naturally occurring compounds and biopolymers as wound healing promoters with antibacterial activity. The emerging approaches and engineered devices for effective wound care in diabetic patients are reported and deeply investigated.

## 1. Introduction

**Figure 1:**
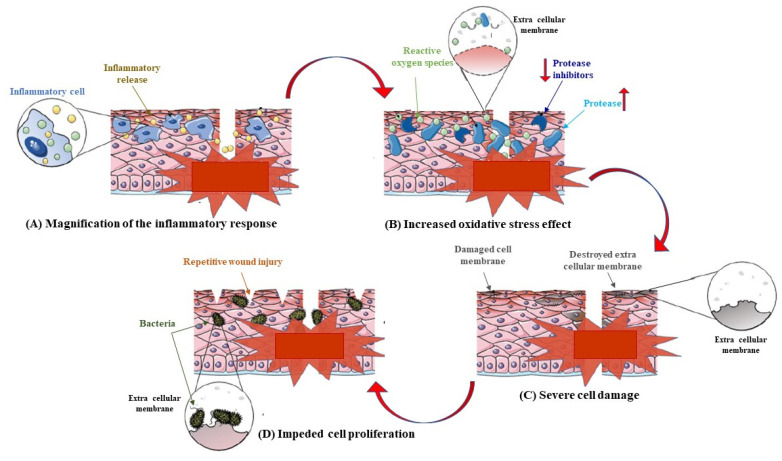
The harmful inflammatory cycle of chronic wounds. During chronic wounds, the anomalous accumulation of inflammatory cells in the wound produces an amplification of the inflammatory response. leading to the destruction of the extra-cellular membrane and the degradation of growth factors and their receptors. The ROS production is then intensified, disrupting the redox balance of cells and enhancing the degradation of the extra-cellular membrane. A delay in the wound healing increases the risk of wound infection [3].

The skin is the largest organ of the human body and the first stage of defense that we have against the external world. Injuries (for example derived from trauma, diabetes, and cancer), burns, and illness can ruin this barrier, leading to wounds. Depending on its severity, wounds can be recovered in a few days (these are named acute wounds) but, sometimes, the complete wound healing process may be influenced by various events such as microbial infection or the presence of free radical products originating from cells of the immune system. In this case, the healing process is prolonged, taking to chronic wounds and therefore to chronic inflammation and chronic pain [1]. Obviously, this translates into the abuse of anti-inflammatory drugs with all their collateral effects [2]. In fact, during the healing process, chronic wounds are unable to timely form a well-organized structure, as it usually takes place in normal healing. This can be considered as a consequence of the severe imbalances usually occurring in the regulation of proinflammatory factors, as schematically reported in Figure 1 [3].

Specifically, the detrimental inflammatory cycle of chronic wounds produces a prolonged inflammatory stage that is responsible for an anomalous increase of the inflammatory cells in the wound. This magnification of the inflammatory response advances with the damage of the extra cellular membrane and the degradation of the growth factors and their receptors, intensifying the production of reactive oxygen species, which alters the redox balance of epidermal and dermal cells, generating a persistent infection. Additionally, this delay in the wound healing process significantly enhances the possibility of wound infection, triggering wound deterioration. Wound infection produced by disproportionate local inflammation significantly delays the wound-healing time, with a negative effect on wound repair. Additionally, *E. coli* bacteria drastically decrease the concentration of collagen type I and collagen type III in the wound, concurring in the delay of the healing of infectious wounds [4]. We all know that the natural microbiota of the skin includes bacteria, viruses, and fungi which create a complex ecosystem that is essential to protect our body against pathogens. When the skin barrier is wounded, the natural bacteria that populate our skin become pathogens. That said, it is obvious that molecules that can simultaneously exert anti-microbial properties and a good wound reparation activity lead to a more efficient wound healing process.

The onset of chronic wounds is an urgent complication for example in patients with diabetes, which is a condition in which higher blood glucose levels drastically impact on the differentiation and proliferation of the cells, as well as new blood vessel formation, leaving the wound in a state of hypoxia and nutritional deficiencies [5]. Usually, diabetic wounds mainly exhibit a no-balanced inflammatory response [6], high blood glucose level [7], absence of angiogenesis [8], oxidative stress effect [9], and a particularly elevated chance of a dangerous bacterial infection occurring [10]. Passive dressings such as bandages and gauze represent useful tools used to avoid wound infection by absorbing wound exudate [11]. However, they are often inefficient because they are unable to fight the continue variations in the wound environment [12]. In this regard, the outline of moist wound healing theory allows for the synthesis of high engineered and modern responsive dressings able to meet the people’s requirements for diabetic wound healing [13]. In fact, innovative functional wound dressings, such as hydrocolloids, nanofibers, and hydrogels, display considerable beneficial effects on the treatment of the diabetic wounds [14]. In particular, hydrogels have attracted much attention due to their strong water absorption performance, as they are able to keep the wound surface environment wet [15]. Additionally, they provide evident improvements in terms of biocompatibility, therapeutic loading capability, and water vapor permeability, ensuring a balanced and useful growth environment for cell proliferation [16]. Considerable interest in the last years has been shown in the use natural polymer as starting materials to produce innovative smart devices [17]. In particular, the involvement of polysaccharides in crosslinking reactions represents a versatile strategy in the manufacturing of intelligent and biodegradable tridimensional systems largely used in the pharmaceutical and biomedical fields [18,19]. Polysaccharides-based hydrogels were widely employed for the fabrication of biological networks, due to their undoubted biocompatibility and biodegradability, as well as non-immunogenicity, water affinity, and non-fouling features. Furthermore, multiple chemical functionalities, such as acid, amine, aldehyde, and hydroxyl groups, can be easily modified by targeted chemical reactions to synthesize a plethora of devices [20]. Animal sources and renewable plants represent the main source of the polysaccharides employed in the pharmaceuticals field, including plants (e.g., agarose, cellulose), microbes (e.g., dextran), algae (e.g., alginate), and animals (e.g., chitosan, hyaluronic acid). Chitosan, cellulose, alginate, and hyaluronic acid are largely employed in the preparation of antibacterial dressing, and are useful in diabetic chronic wound treatment [21]. Different synthetic strategies have been explored in the preparation of the active devices [3]. The polysaccharide-based tri-dimensional matrix can act as polymeric carrier for a specific therapeutic agent and/or themselves are able to preserve the wound from the adverse events, actively contributing to the healing process. In diabetic wound healing, polysaccharides-based hydrogel can perform their antibacterial action by involving a different mechanism strictly related to the network composition, its physicochemical and mechanical properties, as well as the action mechanism of the loaded therapeutics (if any). Sometimes, the diabetic wound healing process is the results of multiple mechanisms able to prevent the invasion of pathogenic bacteria [22], as well as to generate an adequate anti-inflammatory response [23] or to control the level of the reactive oxygen species [24]. Additionally, the promotion of angiogenesis [25] and the avoidance of high levels of blood glucose [26], represent useful mechanisms usually associated with the antimicrobial action. However, a radical distinction cannot always be done, and the multifunctional hydrogel dressings improve diabetic wound healing by a synergistic action of the different mechanisms [27].

Likewise, in the phytochemistry world, a lot of plants (for example Mediterranean plants) were used for a while to cure wounds and skin injuries [28]. During the years it has been demonstrated that these wound repair properties are often ascribed to phenolic compounds [29]. Polyphenols are a large family of secondary metabolites of plants characterized by the presence of a huge number of hydroxyl groups attached to aromatic rings. These molecules are mainly divided into flavonoid and non-flavonoid compounds. Phenols can promote epithelization [30], increase angiogenesis and vascular genesis [31], modulate inflammatory characters such as cytokines [32,33], and ameliorate wound contraction rates [34]. The wound healing process is generally divided into four phases: phase 1, which is hemostasis, phase 2, which is inflammation, phase 3, which is proliferation, and phase 4, which is called remodeling [35]. These phases must take place in an accurate sequence, at a specific time, and be endured for a precise duration in time. Polyphenols most likely have a role especially in the hemostasis phase; in fact, the polyphenol scaffold possesses tissue adhesion properties that can improve hemostatic effects. Some polyphenols moieties such as catechol or pyrogallol groups can interact with serum proteins in the blood, forming complexes that can stop bleeding by creating a physical barrier. All these hemostatic effects can also be due to the antibacterial properties of polyphenols. In addition, phenolic compounds have an efficient light-heat conversion. This latter is important in antibacterial therapy since they can be used as photothermal agents against pathogens [36].

In this review a series of phenolic compounds (Table 1) and biopolymers (Table 4) were researched to discover their wound-healing potential activity and to demonstrate the amazing synergistic effect that natural compounds and nanotechnology can exert. To find the articles, we used several scientific websites such as PubMed, Google Scholar, Science Direct, MedLine, and Reaxys, considering the discoveries of the last 5 years. For the research we used keywords such as: wound healing, antibacterial, anti-inflammatory, and tissue repairing associated with the name of the mentioned compounds and polymers.

## 2. Phenolic Compounds

### 2.1. Flavonoids

#### 2.1.1. Quercetin

Among phenolic compounds, a molecule that possesses wound healing properties associated with anti-bacterial and antioxidant activity is quercetin. The anti-bacterial and wound healing properties of quercetin were evaluated using Quercetin-3-O-A-L-Rhamnopyranosyl-(1→6)-β-D-Glucopyranoside Isolated from *Salvia Leucantha*. Antibacterial activity was tested on some gram-positive bacteria such as *Staphylococcus aureus (S. aureus)*, *Bacillus subtillis (B. subtillis)*, *Sarcina lutea*, and gram-negative *Escherichia coli (E. coli)*, *Pseudomonas aeruginosa (P. aeruginosa)*, and *Candida albicans* (*C. albicans*), comparing data collected with standard antibiotics such as Novobiocin and Chloramphenicol. Quercetin glycosides showed the maximum zone of inhibition compared with standard antibiotics, followed by the quercetin glycosides extract, which was more aggressive against *S. aureus* and *E. coli*, even if the glycosides did not have an effect on *C. Albicans*. At the same time, wound healing capacity was evaluated on wounded healthy albino rats (Wistar strain). Animals were divided into three groups of six rats per group. Group I’s animals were used as a control; the isolated drug was administered topically to animals of group II every day while standard soframycin ointment was administered to group III for 16 days, respectively, as a standard control. Wound areas were measured on days 0, 4, 8, 12, and 16 for all the groups. Data were collected in the following table (Table 2) in order to demonstrate how quercetin treatment accelerates wound closure at almost the same time as standard therapies [37].

A lot of other studies and tests were carried out to evaluate the wound-healing capacity of quercetin. For example, in a study, a formulation of 0.1% ointment in paraffin of the flavonoid was monitored in a time-dependent test on open excision wounds in adult Winstar Rats. The granulation/healing tissue was monitored after 3, 7, 11, and 14 days post wounding with histology/immunochemistry studies done on the growth factors and citokines that are mostly involved in the wound healing process. Groups treated with quercetin showed an improvement in the reduction of the wound area after 7 days and after 14 days the wound was significantly healed. For what concerns immunochemical data, the expression of VEGF and TGF-β1 was significantly improved in the quercetin-treated groups compared with the control group [38].

The effect of quercetin was also evaluated on hypertrophic scars. The direct outcome of an inaccurate wound-healing process is the formation of scars and keloids. Quercetin, administered topically as a cream, was used to cure hypertrophic scars due to excisional wounds done on rabbits. The application of quercetin cream (with 7.5 mg of quercetin on 100 mg of product) three times daily for four weeks leads to a decrease in the hydroxyproline level. The latter was associated with the production of collagen, which is essential in the wound healing process, but there is often a hyper production of collagen that takes keloids and unhealed scars. In addition, quercetin leads to a threefold decrease in the level of histamine compared with the group control, which was administered a placebo cream. So, its effect can most likely be associated with its anti-inflammatory activity (it is able to stabilize mast cell membranes and to inhibit histamine release from basophils and mast cells, especially in the condition of protracted glutathione depletion) but also to its anti-apoptotic effect, thanks to the inhibition of insulin-like growth factor 1 and to the fibroblast proliferation and collagen synthesis [39]. The flavonoid can exert this activity alone or combined with other phenolic compounds, such as curcuminoids. A study evaluated the wound healing capacity and antibacterial activities of quercetin mixed with curcuminoids. The disc diffusion test was used to test the activity against *S. aureus* and *P. aeruginosa*. The best results were obtained for the quercetin/curcuminoid mixture at a ratio of 1:1 On the contrary, quercetin or curcuminoids taken as single compounds did not show antibacterial activity against these two strains of bacteria. This synergistic effect was associated with the two different modes of action of these phenolic compounds to kill bacteria. Quercetin inhibits cell wall synthesis, DNA gyrase, and impairment of cell motility. Curcumin kills bacteria by binding with the FtsZ protein. To test antioxidant activity we used DPPH and ABTS assays and quercetin exhibited the highest DPPH and ABTS free radical scavenging activities. To evaluate the wound healing potential, we tested the ability of these compounds to induce HDFB cell migration in comparison to DMSO 1.25% in serum-free DMEM, which was taken as solvent control in a scratch assay. Human dermal fibroblast cells have an essential role at the re-epithelization stage of the wound healing process. With the cell viability assay we demonstrated that quercetin possessed the highest IC_50_ value against fibroblasts. On the contrary, curcuminoids have higher cytotoxicity against human dermal fibroblasts in a dose- and time-dependent manner. In fact, an increased ratio of curcuminoids in the mixture leads to increased cytotoxicity because it has been demonstrated that curcuminoids induce ROS generation and therefore fibroblast apoptosis as a consequence These data demonstrated the synergistic cytotoxic effects of the mixture of quercetin and curcuminoids against human dermal fibroblast cells [40].

**Figure 2:**
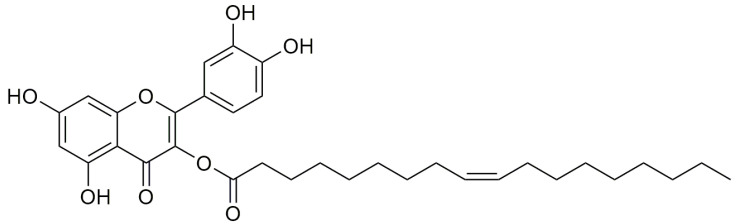
AV2: Quercetin’s oleoyl derivative.

The functionalization of quercetin often leads to more useful compounds. An example can be the oleyil-derivative of the flavonoid, called AV2 (Figure 2), which shows an amazing effect in the wound healing process [41]. AV2 was obtained from quercetin and oleic acid using a green pathway [42]. In order to attempt its mechanism of action several in vitro studies were performed. Previous studies demonstrated that AV2 is a GPR40 agonist [43] and it is well known that G-protein-coupled receptors (GPCRs) are involved in the wound healing process with the modulation of various signaling pathways [44]. In this study quercetin alone was used as a negative control. AV2 was compared with quercetin alone, oleic acid alone, and the molecular hybrid resulted in being more potent. In fact, the cell proliferation rate was improved by 20%, even at the lowest concentration (0.1 μM). Meanwhile, 50 μM of AV2 caused the highest induction of cell proliferation (improved by 40%) compared with the other treatments.

#### 2.1.2. Curcumin

Curcumin is used in a lot of traditional medicines to cure wounds. Its wound healing capacity can be due to a lot of the properties of this compound. First of all, curcumin is an antioxidant molecule, it can reduce reactive oxygen species (ROS), but it has also antibacterial activity against a lot of gram-positive and gram-negative bacteria. Curcumin-encapsulated PEGylated nanoliposomes has a potential anti-infective effect [45] and it also possesses anti-biofilm and antibacterial properties against *Porphyromonas Gingivalis* [46]. Curcumin also has anti-inflammatory properties; for example, it can decrease the production of tumor necrosis factor-alpha (TNF-α), interleukin-1 (IL-1), and nuclear factor kappa-light-chain-enhancer of activated B cells (NF-(κ)B). To demonstrate that the antibacterial activity of curcumin is useful in the wound healing process, some curcumin cross-linked with chitosan-PVA membranes were studied. A total of 10, 20, and 30 mg of curcumin associated with chitosan-PVA was evaluated on wounded rabbits and at the same time curcumin antibacterial activity was assayed in a panel of gram-positive and gram-negative bacteria using the agar disc diffusion method, and the antibacterial results were synthesized in the following table (Table 3).

Data pertinent to the wound healing process were collected on 7th and 14th day of treatment. The wound sizes of animals were measured with a scale, and it was demonstrated that, after 7 days, 30 mg of curcumin/chitosan/PVA accelerated the wound closure process (when applied twice a day), followed by 20 mg of the combination [47]. For what concerns wound healing, curcumin promoted collagen production and enhanced cellular proliferation at the wound site. It also enhanced cytokine production and migration of the fibroblasts to migrate to wound sites, promoting fibroblast and collagen proliferation, supporting complete epithelial repair, and angiogenesis during the proliferative phase of wound healing. All these events lead to faster wound closure. Unfortunately, since curcumin has low solubility and low permeability in vivo systems for its chemical characteristics, a lot of nano-delivery systems were designed and created to improve its activity. In the field of wound healing research, a lot of curcumin-loaded nano formulations were tested, such as nanofibrous membrane with chitosan, gelatin, and PLC or nanoparticles CUR-loaded with chitosan/PEG/silver. All these formulations improve the ability of curcumin to heal wounds. These systems demonstrated very good results in in vitro and in vivo assays and can also have an application in important pathologies such as Diabetic Foot Ulcers (DFUs), a consequence of diabetes that has no real therapy and often can be resolved only with amputation [48].

#### 2.1.3. Pinocembrin

**Figure 3:**
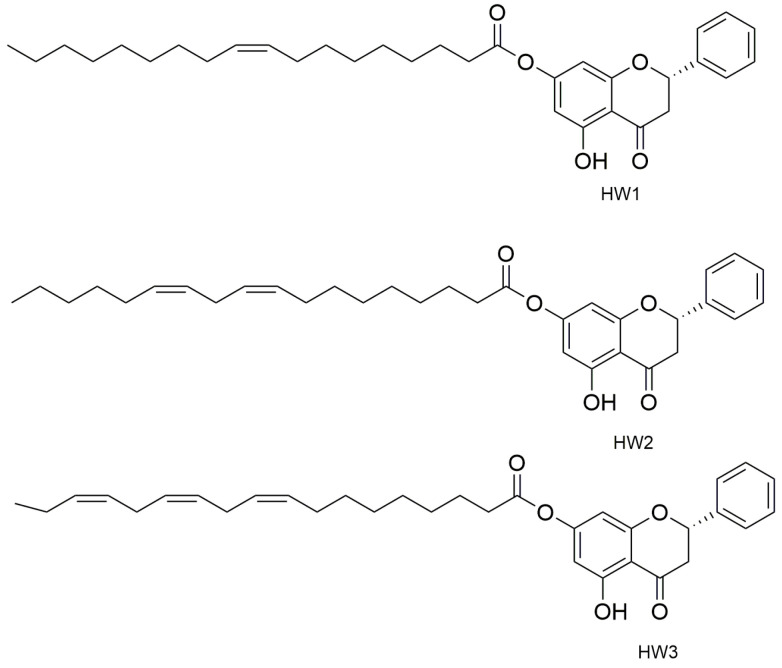
Pinocembrin’s derivatives.

Other players that have demonstrated to play a big role in the skin wound healing process are G-protein coupled receptors (GPCRs). They are activated through the decrease of pH (a phenomenon that often occurs in the wound) and this is transduced to a molecular intracellular pathway that converges in the tissue repair. To demonstrate this, the endogenous ligand of GPCRS, docosahexaenoic acid (DHA), promoted wound healing when targeting GPR120 in vitro and in vivo. A molecule that is an agonist ligand for GPR120 is pinocembrin, which is a flavone with great anti-bacterial properties. The flavone and some of its derivatives (Figure 3), oleoyl-HW1, linoleoyl-HW2, and linolenoyl-HW3 esters, were studied to test their wound healing capacity firstly in an in vitro scratch wound healing assay to evaluate the keratinocytes, with the transforming growth factor β (TGF-β) as a positive control. Pinocembrin was able to stimulate HaCaT wound healing after 6 and 24 h (about +30% compared to the untreated control) in a concentration-dependent manner. Some derivatives such as HW1 and HW2 were less active than pinocembrin, but data carried out from HW3 were interesting. The wound-healing activity of pinocembrin was also demonstrated in some tests carried out in vitro but with the antagonist of GPR120 (AH7614). To understand the mechanism of action of the flavone in the process, we hypothesized that GPR120 activation takes to an increase in TGF-β, which stimulates the synthesis of extracellular matrix components, essential with keratinocytes for the wound healing process. In addition, an increase of IL-1β release was observed in THP-1 cells treated with 1 µM HW3, while the highest concentration of HW3 stimulated only the release of TNF-α. Cytokines also take to metalloproteinases (MMPs) production, and some enzymes that are important for extracellular matrix degradation are essential for wound healing, especially for MMP-9, which has a key role in the rephitalization process. In this study, HW3 takes to an increase of MMP-9 levels even if HW0 did not modify MMP-9 levels [49].

Pinocembrin is also the main flavonoid component of a lot of species of honey. This molecule and its 7-methylether derivative, extracted from two types of Calabrian honeys, were tested in vitro to determine their wound healing properties. In fact, these two types of honey, which are called BL1 (a multifloral honey) and BL5 (an orange honey), showed an increase in the wound healing rate in a scratch wound-healing assay on HaCaT cells, enhancing the cell migration. BL1 was active at each of the tested concentrations, even at the lowest (0.1 mg mL^−1^), increasing the wound-healing rate by 27, 51, 47, and 52%, compared to the untreated control. Instead, 1 mg mL^−1^ and 10 µg mL^−1^ of BL5 enhanced the wound-healing rate by 61 and 22%, respectively. H-NMR and gas chromatography assays have shown the presence of pinocembrin in all the active kinds of honey, so even the flavone was tested in vitro. This latter increased the wound-healing rate by 25% at every tested concentration. In addition, cells proliferation assays were performed for every sample, but the results were negative, confirming the stimulation of keratinocytes’ migration as the wound healing mechanism. This activity may be very useful to treat wounds such as diabetic wounds, in which there are altered cytokines and growth factor levels and reduced keratinocytes and fibroblast migration [50].

#### 2.1.4. Chrysin

Another phenolic compound that has been demonstrated to act as a potent stimulator of keratinocyte differentiation in accelerating the natural wound healing process is the phenol chrysin. To stem chrysin’s poor bioavailability, some alginate-chitosan-chrysin loaded composites were designed and created. These scaffolds were used to demonstrate the wound healing capacity of chrysin and tests were carried out on healthy adult male Wistar rats, and chrysin was used at doses of 5,10, 20, 30, and 40 μM for 24 h, 48 h, and 72 h. Animals were divided into three groups: group 1 was the control and contained animals dressed with non-medicated medical gauze, group 2 contained animals dressed with Alginate-Chitosan scaffold (ALG-CS), and group 3 with animals dressed with Alginate-Chitosan Chrysin loaded scaffolds (ALG-CS-CHY). In summary, the rate of wound contraction was higher for the third. In addition, hydroxyproline analysis was performed to quantify the collagen with Masson’s trichrome-stained sections of the granulation tissue. After only 4 days, the collagen synthesis and deposition were higher for the third group, and on the 8th and 12th days, there were a lot of new collagen fibers with dense collagen deposition [51]. In another study, chrysin was associated with curcumin, creating some chrysin–curcumin-loaded nanofibers active on the wounded male rats. The wound area was controlled after 5-, 10-, and 15-days post-injury for all the groups. The rate of the wound closure was higher than the controls even if the activity was dose dependent; in fact, the chrysin-loaded nanofiber significantly improved wound-healing process at higher doses. Regardless, the wound closure efficacy of the nanofibers containing curcumin was higher than that of chrysin, and the difference was not significant. Data showed that there is no synergistic effect between chrysin and curcumin for wound closure. The wound healing activity of chrysin–curcumin-loaded nanofibers can be due to their ability to modulate some factors involved in the inflammatory processes that are essential for a good wound healing process. For example, nanofibers were able to elevate the expression of IL-6, MMP-2, TIMP-1, TIMP-2, and on the contrary, they reduce the expression of the iNOs gene. The results demonstrated that the effect of nanofibers in the wound-healing process is dose-dependent, and it can influence the inflammation phase more than the other stages of the wound-healing process [52].

#### 2.1.5. Luteolin

Among polyphenols that possess antibacterial activity, there is Luteolin. Luteolin is considered a natural antibiotic because it can destroy the cell membrane of bacteria, inhibit nucleic acid synthesis, and modulate protein expression and energy metabolism. However, as with most flavonoids, luteolin itself has poor solubility and poor biodisponibility. So, to assess its antibacterial and wound repairing capacity, some chitosan–indocyanine green/luteolin nanocomposites were created and tested. In the study, nanoplatforms’ activity was induced by near-infrared irradiation to improve their activity thanks to the photothermal/chemotherapy. Antibacterial tests were carried out on a *S. aureus* strain to quantify the disruption of the bacterial membrane used for the ONPG hydrolysis assay. Some antibacterial assays were conducted in vivo on male Balb/c mice during in vitro antibacterial studies, NIR application alone showed no relevant effects on *S. aureus*, highlighting the bacteriostatic effect of luteolin. Ten µg mL^−1^ of the ICG/LUT-CH composites treatment group led to a bacteria reduction of up of 50% and with the NIR treatment, the bactericidal effects of ICG/LUT and ICG/LUT-CS against *S. aureus* were significantly improved (40 µg mL^−1^ could reach almost 100% of bacteria reduction). Antibiofilm formation capacity was also examined by the CV staining assays. Once again, NIR treatment itself showed no effect on biofilm formation while the ICG/LUT and ICG/LUT-CH treatment groups demonstrated a significant antibiofilm effect, demonstrating the effect of luteolin. In the end, wound healing capacity was assessed on a *S. aureus* infected skin wound model. When the ICG/LUT was applied, the wound region temperature increased rapidly from 35 to 52.6 °C, demonstrating the photothermal activity of nanocomposites. The macroscopic investigations showed that in the wound area in which the treatment was applied, there were no signs of ulceration and suppuration. ICG/LUT-CS with the NIR group exhibited a superior wound-healing rate and a major wound area reduction. On Day 8, there was wound tissue on the epithelial layers in all the treatment groups but in the control we observed an infiltration of inflammatory cells with a severe bacterial infection, while the ICG/LUT-CS+NIR group had fewer inflammatory cells, new hair follicles, new blood vessels, and no severe bacterial infection, demonstrating the efficacy of luteolin and of this treatment against pathogens but above all in the wound healing process [53].

#### 2.1.6. Catechin and Epigallocatechin-3-Gallate (EGCG)

One example is the latex of the plant *Jatropha neopauciflora Pax*, an endemic species of Mexico used to heal wounds. It has antibacterial and bacteriostatic activity against Gram-positive bacteria, particularly *S. aureus* (with a MIC = 2 mg mL^−1^). the Kirby–Baüer disc diffusion agar method was used to determine the antibacterial activity, while the tensiometric method, based on measuring wound resistance to tension, was used to test wound healing capacity. The collected data were considerable even when compared to the positive control Recoveron, an unguent commercially available wound treatment. For anti-inflammatory tests we used the carrageenan-induced edema model (100 μL, 1%, dissolved in NaCl, 0.9%) on Wistar rats, and, to determine the antioxidant properties of the compounds, we used a DPPH test. Latex was able to reduce DPPH thanks to the concentration of phenols (6.9 mg GA mL^−1^) and flavonoids (12.53 µg Q mL^−1^). To calculate phenolic content we first used the Folin-Ciocalteu assay, which gives back information about the total phenolic content of latex, while The Dowd method was used to determine the content of flavonoids. HPLC analyses conducted in the end on the latex highlighted the presence of some phenolic compounds such as catechin and epigallocatechin-3-gallate (EGCG) [54].

### 2.2. Tannins

Among phenolic compounds, another class, the tannins, showed good wound-healing properties associated with antibacterial activity. Tannins can enhance the wound healing process through various mechanisms, for example the chelation of free radicals and reactive species of oxygen, the promotion of the wound’s contraction and improving angiogenesis and fibroblasts’ formation, and the ability to complex with proteins at the injury site, forming a protective layer on the injured zone [55]. Tannins extracted from the fruits of *Terminalia chebula Fructus Retz.* demonstrated good antibacterial properties that were directly associated with good wound healing properties. Antibacterial activity was tested using the micro-dilution method, with antimicrobial drugs such as penicillin or cefoperazone sodium as the control. Studies were done both on gram-positive bacteria such as *S. aureus* (with MIC value 0.3125 mg mL^−1^ and MBC value 1.25 mg mL^−1^) and both against gram-negative bacteria such as *Klebsiella Pneumonia* (with MIC value 0.3125 mg mL^−1^ and MBC value 0.625 mg mL^−1^). Wound healing capacity was calculated by measuring planimetrical wound areas after the excision on three groups of rats. The first group was used as a negative control with paraffin oil ointment, on the second group we administered tannins and the third group was the positive control with erythromycin ointment. The final results demonstrated that the percentage of wound contraction was higher for groups II and III after 7 and 10 days. One important part of wound healing process is revascularization, and this process is regulated also by the endothelial cell growth factor (VEGF). So, in the study, the VEGF-A expression was also calculated and this latter was higher on the third day only for groups II and III. Tannins also demonstrated that a decrease in the permeability of the capillaries in the wound alleviates tissue edema and exudation [56].

#### Tannic Acid

Tannic acid (TA) is a specific form of tannin, a type of polyphenol. It was used in the first decades of 1900 for the therapy of burn wounds. This polyphenol was demonstrated to have antibacterial activity against a lot of bacterial forms, for example *S. aureus*, *E. coli*, or *P. aeruginosa*. The antibacterial activity of TA is exerted though various ways, such as the destabilization of the cytoplasmic membrane, the improvement of the permeability of the membrane, and the inhibition of enzymes. To demonstrate its wound healing properties, tannic acid was combined with zinc salts and carboxylated agarose to obtain new pH-responsive hydrogels. This idea came to be, because even if the pH of skin has a steady value of 5.5, during the wound formation processes various mechanisms influence the pH. The antibacterial activity of hydrogels was tested against S. Aureus in agar disc diffusion test and analysis revealed that TA has the same antibacterial activity of gentamicin. Wound healing properties were demonstrated though cells migration tests. Assays enlightened that TA promotes cell proliferation and migration, which are processes that are essential in wound repair [57].

### 2.3. Terpenoids

#### 2.3.1. Terpinolene and α-Phellandrene

Terpenoids are a class of polyphenolic compounds. Ninety percent of them are essential oils. Among these, a lot of molecules exert wound-healing activity. For example, two monoterpenes with bioactive properties such as antibacterial properties, exhibited wound repairing capacity in scratch assays conducted on artificial cell lines to test the proliferation and migration of fibroblasts. Data showed that 200 μM of both molecules improved the proliferation and migration of fibroblasts compared to the control (untreated cells) in a dose-dependent manner, reaching maximum stimulatory effects of 36.3 ± 4.8% and 39.1 ± 3.9%, respectively [58].

#### 2.3.2. Thymol

Another monoterpene, thymol, showed an emerging importance in the wound repairing processes, thanks to its antibacterial properties. In fact, thymol was active against a huge panel of both Gram-negative (*E. coli*, *Proteus mirabilis*, *Proteus vulgaris*, *Salmonella typhimurium*, *Serratia marcescens*, *Yersinia enterocolitica*, *Pseudomonas fluorescens*, *Pseudomonas putida*) and Gram-positive (*Micrococcus* spp., *Sarcinaflava*, *S. aureus*, *Bacillus licheniformis*, *Bacillus thuringiensis*, and *Listeria innocua*) bacteria [59]. In a study, thymol was obtained from the essential oil of *Lippia gracilis* and incorporated into collagen-based dressing films. Wound healing analyses were done after 3, 7, 14, and 21 days using a digital caliper on three groups of adult Wistar rats considering: undressed wounds (CTR), dressed with collagen-based films (COL), and dressed with collagen-based containing thymol (COLTHY) films. In the COLTHY group, wound size was significantly reduced on days 7 and 14 compared to CTR and COL. In this group, the severity of inflammation processes was low after 7 days. The formation of collagen was analyzed and, even in seven days, the process of collagenization was similar for all groups; only in the COLTHY group were there denser, thick, and parallel-arranged collagen fibers. In 14 days, for the same group, the fibers were more compact and there were fewer interfibrillar spaces, particularly in the marginal areas. Even in 21 days, the fibers in the COLTHY group were gross and thicker, very similar to the normal dermis. In addition, edema was reduced [60].

### 2.4. Alkaloids

#### Taspine

Another compound that showed unexpected wound-healing properties is taspine, an alkaloid extracted from the trees of Croton. In a study conducted on several groups of male Sprague-Dawley rats, Taspine enhanced the wound healing process in a dose/time-dependent manner. In fact, every group received a different dose of the alkaloid (starting from 10 µg to 250 µg, except for the first group that received only DMSO, and analysis was carried out for different periods). Only the maximum dose of taspine (250 µg) showed wound healing capacity after 5 and 7 days, but for no longer than 12 days. In vitro histological assays were performed, and, in the end, the alkaloid demonstrated to promote fibroblast migration, with an optimal cell migration at 50 pg mL^−1^, showing the chemotactic effect for fibroblasts as its mechanism of action [61].

## 3. Polymeric Compounds

In Table 4, the chemical structures and main physicochemical and biological properties of the most employed polysaccharides in the treatment of the diabetic wounds are reported.

### 3.1. Chitosan-Based Hydrogels

Chitosan (CH), a natural co-polymer derived from chitin, shows excellent antibacterial properties and biodegradation, and it is useful in hemostatic applications such as wound adhesive biofilms and hydrogels and is very promising in diabetic wound healing [62]. Mainly due to its remarkable antibacterial properties, CH has been largely involved in the synthesis of functional hydrogels useful to prevent bacterial infections and to accelerate the healing of the diabetic wound (Table 5).

However, the antimicrobial effect of CH is often not sufficient due to the complicated pathological mechanism of diabetes mellitus and the cooperation of other active substances is required to ensure rapid healing of diabetic wounds. Biopolymers are often used to transport and deliver metal nanoparticles, widely employed to accelerate the healing of wounds [63]. In particular, silver nanoparticles are characterized by a wide spectrum of antimicrobial activity, but their use in biomedicine is still limited by toxicity problems [64]. Thus, the adducted metal nanoparticles-polysaccharides leads to a significant reduction of the toxicity and an enhancement in biocompatibility with living tissues [65]. Silver nanoparticles loaded CH-Polyethylene glycol (PEG) hydrogel were developed for diabetic wound healing application [66]. PEG is approved by FDA for different pharmaceutical and biomedical requests, and is useful in the healing of chronic wounds in diabetes to increase non-immunogenicity and water solubility of the polysaccharide chain [67]. A slow release of silver nanoparticles from CH-based hydrogel was recorded over a period of 7 days, while the antimicrobial activity against *P. aeruginosa*, *E. coli*, *S. aureus* and *Bacillus subtilis* revealed a higher reduction rate in viable bacterial counts. Additionally, the hydrophilic material also displayed relevant antioxidant capacity, as confirmed by scavenger activity against 2,2′-diphenyl-2-picrylhydrazyl radical (IC_50_ of 129.5 μg mL^−1^). Silver nanoparticles were also effectively transported by a gel synthetized involving CH and collagen [68]. It is a fibrillar protein able to accelerate the regeneration of tissue and skin cells. It is usually crosslinked with polysaccharides macromolecules to overcome the issues associated with its poor mechanical properties and rapid degradation [69]. Genipin-mediated chemical crosslinking allowed the synthesis of a biocompatible and swellable tridimensional network with remarkable antibacterial (against *E. coli*, *S. aureus* and *Staphilococcus epidermidis*) and antifungal activities (against *C. albicans*).

CH hydrogel for wound healing application was also synthesized by ionic cross-linking involving the hydroxyl and amino groups of the polysaccharide chains and specific metal ions (Ag^+^ and Cu^2+^) with antibacterial and angiogenetic activity [70]. Specifically, Ag^+^ displays remarkable antibacterial ability [71], while copper (II) ions coupled a certain antibacterial ability with a significant capacity to promote collagen deposition and angiogenesis processes [72]. With respect to traditional silver nanoparticles, Ag^+^ ions displayed enhanced antibacterial ability, and their retained release from tailored hydrogel wound dressings overcomes the problems usually associated with the limited period in which it can be consumed [71]. The antibacterial activity of the multifunctional hydrogel was evaluated in vitro against Gram-positive *S. aureus* (minimal bactericidal concentration 4.0 mg mL^−1^) and Gram-negative *E. coli* (minimal bactericidal concentration 2.0 mg mL^−1^), while the angiogenic capacity concurs to the tissue repair (14 days) in a *S. aureus*-infected skin in normal rats and diabetic wounds. Alternatively, a synthetic CH-based composite hydrogel incorporating Ag^+^, and nanoparticle-encapsulating epidermal growth factor was established [73]. A multifunctional hydrogel offers better wound healing capacity by accelerating the re-epithelialization and collagen deposition against diabetic wound. Interestingly, in this study, angiogenesis and antimicrobial (against *S. aureus* or *S. epidermidis*) mechanisms concur to the wound healing process, reaching a degree of wound closure of 97% after 14 days. Similarly, antibacterial, and anti-inflammatory activities were analyzed in the evaluation of a multifunctional CH-based hydrogel loaded with Ag nanoparticles and *Calendula officinalis* L. [74]. This device was proposed in the treatment of patients with diabetes type II and the recorded results highlighted a significant improvement in both healing on vascular injuries, due to the ability of the patch to stimulate fibrinolysis and healing and reducing the possibility of infection. Similarly, the active molecules of the *Pterocarpus marsupium* heartwood extract were effectively loaded on chitosan nanoparticles, and were subsequently involved in the preparation of different carbopol hydrogel formulations [75]. The extract represents a rich source of bioactive substances, which can play an important role in the anti-inflammatory and antioxidant response. The prepared wound dressing material was discovered to be non-toxic and non-allergenic, retaining the skin moist absorbing wound exudates, and protecting the wound from microbial organisms. In vivo experiments performed on diabetic rats displayed 100% of wound closure after 18 days.

**Figure 4:**
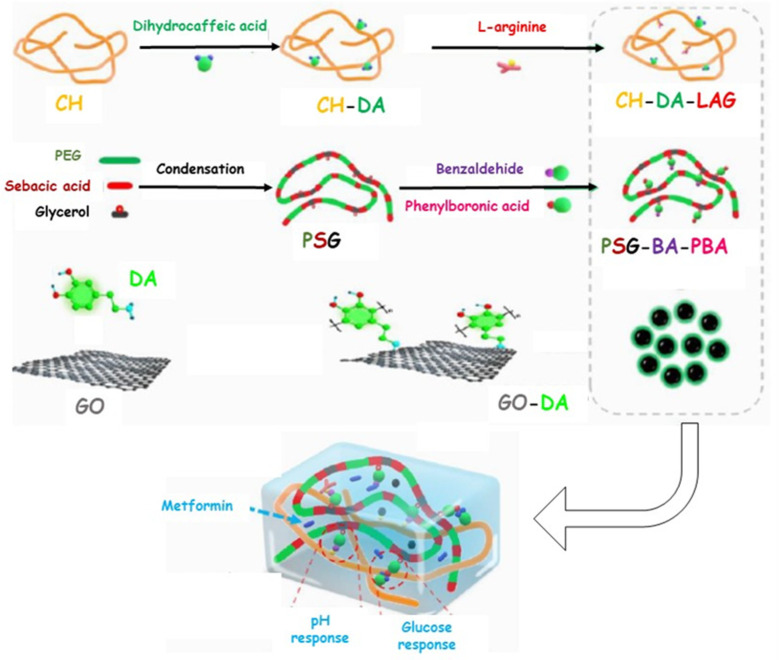
CH-based multifunctional hydrogel for diabetic wound treatment. Preparation of dihydrocaffeic acid (DA) and L-arginine (LAG) co-grafting chitosan (CS-DA-LAG) and phenylboronic acid (PBA) and benzaldehyde (BA) difunctionalized polyethylene glycol-co-poly(glycerol sebacic acid) (PSG) and coated graphene oxide (GO-DA) [78].

Otherwise, polyphenol molecules can be successfully involved in the chronic wound healing process by preventing the oxidative stress generated from the intense inflammatory response. In this regard, the antibacterial and antioxidant properties of curcumin were exploited by loading the active molecule into a CH-based hydrogel synthesized by ionic crosslinking [76]. Alternatively, the CH-based hydrogel was in situ formed by means of Horseradish Peroxidase-catalyzed oxidative crosslinking of pyrogallol moieties and proposed in the treatment of diabetic wounds. Similarly, methacrylate-CH was grafted with gallic acid to produce a conjugate with outstanding antioxidant features [77]. Subsequently, a multifunctional hydrogel was synthesized by loading the F127/chlorhexidine nanoparticle, which is able to confer antibacterial proprieties to the device. In vivo results proved that the antimicrobial and antioxidant properties of the loaded hydrogel significantly reduced the inflammatory response, promoting angiogenesis and enhancing the collagen deposition and tissue re-modelling in the diabetic wound healing. A multifunctional hydrogel was prepared, involving di-hydrocaffeic acid and L-arginine co-grafted CH (CH-DA-LAG) in a crosslinking reaction via the double dynamic bonds of a Schiff base and dynamic phenylboronate ester with phenylboronic acid and benzaldehyde bifunctional polyethylene glycol-co-poly(glycerol sebacic acid) (Figure 4) [78].

This system was proposed for the site-specific delivery of Metformin, a commonly used clinical drug for type II diabetes treatment, and polydopamine-coated reduced graphene oxide to afford hemostasis and conductivity. In vivo experiments displayed an endorsing effect on the healing of chronic athletic diabetic wounds, providing a local-specific strategy for the treatment of the diabetic feet. To improve the mechanical properties of the dressing, CH-based hydrogels can be combined with biocompatible, biodegradable, and hydrophilic synthetic polymer, such as polyvinyl alcohol (PVA) [79]. In this regard, PVA/CH hydrogel was loaded with Tibetan 18 flavor *dangshen* pills, a mixture of 18 traditional Tibetan medicines able to reduce inflammation, promote the repair of defects in the skin tissue, and which possess undoubted antibacterial properties and antioxidant activity [80]. The loaded hydrogel displayed excellent biocompatibility and good antibacterial activity against *E. Coli* and *S. Aureus* bacteria, as well as high cell proliferation, and antioxidant properties, able to decrease DNA damage, lipid peroxidation, and enzyme inactivation caused by the oxidative stress. Furthermore, a PVA/CH heterogeneous composite hydrogel containing perfluorocarbon emulsions, epidermal growth factor-loaded CH nanoparticles, and poly-hexamethylene biguanide, was established for diabetic wound healing [73]. The multifunctional system displayed excellent anti-inflammatory and angiogenetic features, as well as improved antimicrobials properties against *S. aureus* and *S. epidermidis*, due to the cooperative action of CH backbones and poly-hexamethylene biguanide. In vivo experiments performed on diabetic rats returned excellent re-epithelization, quicker collagen deposition, and reduced inflammatory response, resulting in a 95% of wound closure degree after 15 days.

Carboxymethyl chitosan (CMCH) represents the most important water-soluble derivative of CH [81] and, with respect to CH, it shows an improved water solubility and oxidation resistance, providing increased application opportunities, due to the enhanced antibacterial properties [82]. Bio-multifunctional benzaldehyde-terminated 4-arm poly(ethylene glycol)/CMCH/basic fibroblast growth factor hydrogels are prepared using the dynamic Schiff base reaction [83]. The antibacterial properties of CMCH-based hydrogels against were tested on both *E. coli* and *S. aureus* and more than 67% of microbial species were killed. The in vivo experiments highlighted that the hydrogel dressing greatly accelerates full-thickness diabetic wound repair by promoting, after 14 days, the generation of epithelialization and collagen and enhancing the neovascularization processes. CMCH was successfully crosslinked with polyvinylpyrrolidone-iodine (PVPI), an ionophore classified as clinical antiseptics with widespread use in clinical applications for more than 20 years [84]. The cooperative antibacterial action of CMCH-PVPI hydrogel was assessed against *S. aureus* bacteria, while in vivo experiments on diabetic wounds revealed a significant acceleration in the wound closure (14 days).

Quaternized chitosan (QCH) is a partial derivative of CH, showing outstanding solubility, and biodegradability. It is frequently used as raw biopolymer to construct antibacterial materials, due to its higher antimicrobial activity and improved water solubility compared to native chitosan [85]. Innovative hydrogel dressing with outstanding scavenging property and significant antibacterial performance against both gram positive and negative bacteria was synthesized by introducing tannic acid into QCH polymeric matrix [86]. QCH/tannic acid adduct rapidly inhibits the growth of *E. coli* and *S. aureus*, also displaying scavenging capability and is able to protect the diabetic wound from the reactive oxygen species. In vivo wound healing on hydrogel treated diabetic rats showed faster collagen deposition and enhanced skin tissue regeneration after 15 days. Cheng and co-workers exploited QCH and benzaldehyde-terminated 4-arm poly(ethylene glycol) as raw materials to form tridimensional network by in situ gelation reaction also involving ε-poly-L-lysine grafted graphene quantum dots [87]. The synergistic antibacterial effect of QCH and modified graphene was explored against *E. coli*, *S. aureus*, and *P. aeruginosa* bacteria and, in all cases, huge damage to the inner membrane was recorded. Antibacterial conductive hydrogel was constructed by grafting poly-(N-acryloyl glycinamide) and polyaniline onto QCH [88]. The synergistic action of the polyaniline segments and quaternary ammonium groups of CH displayed intense antimicrobial activity against *P. aeruginosa* and *S. aureus* biofilms, with bactericidal ratios of over 80%. Additionally, in vivo tests in diabetic rats demonstrated that the electrical stimulation via the conductive hydrogel was more effective in endorsing the healing of infected wounds than the conventional electrical stimulation via rigid electrodes. More recently, a versatile hydrogel dressing, containing insulin, was synthesized by Schiff base formation between the amino groups on QCH and the aldehyde groups on benzaldehyde-terminated F108 micelles, incapsulating CORM-401, an oxidant-sensitive CO-releasing molecules [88]. A significant in vitro antimicrobial activity against *E. coli* and *S. aureus* species was recorded, mainly ascribed to the combined action of QCH and CO species. The concurring antioxidant and anti-inflammatory mechanisms, as well as the ability to regulate the blood glucose concentration, significantly promoted the healing of Streptozotocin-induced methicillin-resistant *S. aureus*—infected diabetic wounds, reaching a wound contraction rate of 97% after 15 days. More recently, QCH and star-like eight-arm cross-linker octa-functionalized polyhedral oligomeric silsesquioxane of benzaldehyde-terminated polyethylene glycol (POSS-PEG-CHO) was explored in the synthesis and biological evaluation of an innovative antibacterial hydrogel [89]. An in vitro antibacterial test against *E. coli* and *S. aureus* returned a reduction of microbial species equal to 70.0% for Gram-positive bacteria, and 90.5% against the Gram negative one. Finally, wound healing experiments performed on diabetic mice proved that the composite hydrogels had a noteworthy promoting effect on the healing of the diabetic wounds.

**Table 5:** Antibacterial CH-based hydrogels employed in the diabetic wound healing.

Hydrogel Composition		Delivery Properties		Wound Healing		Ref.
Polysaccharide	Component	Bioactive Agent	Concentration	Mechanism	Time (Day)	
CH	PEG	Silver nanoparticle	0.1% (*w*/*w*)	Antioxidant Antibacterial	14	[66]
CH	Collagen	Silver nanoparticles	-	Antibacterial	6	[68]
CH	-	Silver nanoparticles *Calendula* extract	10.9–14.5% (*v*/*v*) 3.6–27.3% (*v*/*v*)	Anti-inflammatory Antibacterial	15	[74]
CH	Carbopol	*Pterocarpus marsupium* heartwood extract	10% (*w*/*w*)	Antibacterial Antioxidant	18	[75]
CH	-	Ag^+^ Cu^2+^	0.37 mol L^−1^ 0.15 mol L^−1^	Antibacterial Pro-angiogenesis	14	[70]
CH	PVAc	Ag^+^ Epidermal growth factor	0–60 μg mL^−1^ 0–96 mM	Antibacterial Pro-angiogenesis	14	[73]
CH	-	Curcumin	0.5–1.5% (*w*/*w*)	Antibacterial Antioxidant	-	[76]
CH	PVA	Tibetan dangshen pills	5–20% (*w*/*w*)	Antibacterial Antioxidant	21	[3]
CH	PVA	Polyhexamethylene biguanide Epidermal growth factor Perfluorocarbon	60 μg mL^−1^ 60 μg mL^−1^ 50 mg mL^−1^	Anti-inflammatory Antimicrobial Pro-angiogenesis	15	[73]
CHMA-g-GA	-	F127/chlorhexidine nanoparticles	0–0.1 mg mL^−1^	Antibacterial Antioxidant	20	[77]
CH-DA-LAG	PEG-co-poly(glycerol sebacic acid) GO-Polydopamine	Metformin	1 mg mL^−1^	Anti-inflammatory Pro-angiogenesis Antioxidant Antibacterial	21	[78]
CMCH	-	Fibroblast growth factor	4200 IU mL^−1^	Antibacterial Pro-angiogenesis	14	[83]
CMCH	PVPI	-	-	Antibacterial	14	[90]
QCH	-	Tannic acid	0.05% (*w*/*w*)	Antibacterial Antioxidant	15	[86]
QCH	ε-poly-L-lysine grafted graphene quantum dots	-	-	Antibacterial	14	[91]
QCH	N-acryloyl glycinamide Polyaniline	-	-	Antibacterial Antioxidant	14	[88]
QCH	F108-CHO	CORM-401 Insulin	1–3% (*w*/*w*) 0.5–1.5% (*w*/*w*)	Anti-inflammatory Antibacterial Antioxidant Anti-glycaemic	15	[87]
QCH	POSS-PEG-CHO	-	-	Antibacterial	21	[89]

CH = chitosan; CH-DA-LAG = dihydrocaffeic acid L-arginine co-grafted chitosan; CHMA = chitosan methacrylate; CMCH = carboxymethyl chitosan; CORM-401 = oxidant-sensitive CO-releasing molecule; GO = Graphene oxide; PEG = polyethylene glycol; POSS-PEG-CHO = functionalized benzaldehyde-terminated polyethylene glycol; PVA = polyvinyl alcohol; PVAc = polyvinyl acetate; PVPI = polyvinylpyrrolidone-iodine; QCH = quaternized chitosan.

### 3.2. Others Polysaccharides-Based Hydrogels

#### 3.2.1. Sodium Alginate (SA)

Sodium alginate (SA) was employed in the treatment of the wounds, due to its ability to promote cellular proliferation and adhesion, to reduce bacterial liability, as well as its remarkable hemostatic properties [92,93]. Additionally, the hydrophilic nature of SA allows the preservation of the physiologically moist wound environment, accelerating the healing cascade of the wound. The limitation of alginate hydrogels, however, is their low and random degradation in vivo, through dissociation of the ionic crosslinking [94]. Injectable hydrogels with multifunctional tunable features were synthesize by the solvent casting method involving SA, Pluronic-F127, and chondroitin sulphate (Table 6) [95]. This system was proposed to transport a delivery loaded curcumin to achieve a device able to endorse a healing cascade by improving re-epithelization and increasing the collagen deposition and angiogenesis at the wound microenvironment. In vitro antibacterial features were evaluated by using *P. aeruginosa*, *S. aureus*, and *E. coli* strains, while the curcumin release in simulating microenvironment wounds was performed by returning a release profile with an initial burst effect and a complete delivery of the bioactive after 24 h. Furthermore, in vivo experiments highlighted the high diabetic wound healing and tissue-restructuring potential of the device with a complete skin tissue regeneration after 20 days.

#### 3.2.2. Hyaluronic Acid (HA)

Hyaluronic acid (HA) is a biocompatible natural glycosaminoglycan and a basic constituent of the extracellular matrix in the skin. It can stimulate the natural extra cellular membrane assembly by interacting with proteins in the physiological environment [96]. It is employed in wound healing due to its capacity to stimulate the angiogenic ability of endothelial cells, increasing the proliferation and migration of keratinocytes and fibroblasts [97]. Additionally, the high molecular weight of HA acts as inflammation regulating by stimulating the conversion of the macrophages from a pro-inflammatory M1 to a reparative M2 phenotype [90], allowing the delivery of anti-inflammatory growth factors and cytokines [98]. Although some HA-based hydrogels are commercially available in clinics (Hylase Wound Gel, Hyalofill), weak mechanical properties, as well as rapid degradation and poor adhesion considerably limited the employment of these raw materials in the treatment of the diabetic wounds [99]. The antibacterial hydrogel based on HA employed in the diabetic wound healing were summarized in Table 2. The injectable and thermo-responsive HA/hexamethylene diisocyanate-Poloxamer 407 copolymer crosslinked hybrid hydrogel consisting of fluorocarbon nanodroplets, epidermal growth factor-loaded nanoparticles, and poly-hexamethylene biguanide (PHMB) was effectively established for the treatment of the diabetic wounds [100]. In particular, PHMB may further increase the antimicrobial effects of the device and various dosages (100–400 ppm) were tested against *S. aureus*. The bacterial population index was significantly decreased by 53% when the dose of PHMB was increased from 100 to 200 ppm, while at an elevated dose (≥400 ppm) none of bacterial colony was detected. The in vivo experiments performed on diabetic rats displayed that the synergistic action (anti-inflammatory, pro-angiogenesis, antioxidant, and antibacterial) of the different components guaranteed a complete wound closure after 15 days.

#### 3.2.3. Cellulose

Cellulose and its derivatives are naturally occurring polysaccharides, usually produced via different fungi and bacteria, and are mainly present in the plant’s cell walls [101]. Hydrogel based on cellulose employed in the diabetic wound healing were summarized in Table 2. Bacterial cellulose (BL) is a kind of biocompatible nano-polymer produced by both Gram-negative and Gram-positive bacteria, mainly *Komagataeibacter xylinus* [102]. BL was largely used as dressing due to its outstanding processing features, high water content, and excellent thermal stability [103]. Additionally, it shows high tensile strength (Young’s modulus reaching 61–95 GPa), good elasticity, as well as a remarkable ability to control wound exudate and maintain a moist wound environment [104]. BL was more effective than gauze in endorsing tissue proliferation with more complete epidermal layers and the formation of compact collagen, and the diabetic wounds of the mice treated with BL healed 1.63 times faster than those treated with gauze [105].

Cellulose acetate (CA), a derivative of natural cellulose, is an important component of the cell wall of green plants and is widely available and inexpensive [106]. A multifunctional wound dressing with remarkable antibacterial features was synthesized using CA as hydrophilic matrix and Dimethyl-oxallyl Glycine and silver nanoparticles as bioactive molecules [107]. The release profile of Dimethyl-oxallyl glycine was recorded in physiological medium and a significant burst effect (52%) in the first experimental time (1 h) was observed, with a complete delivery of the therapeutics after 84 h. The antibacterial capacity of the tridimensional system, evaluated against *E. coli* and *B. subtilis* bacteria, is strictly related to the presence of silver nanoparticles, and ensures biological activity for at least 48 h, while no drug resistance will be produced.

**Table 6:** Antibacterial polysaccharides-based hydrogels employed in the diabetic wound healing.

Hydrogel Composition		Delivery Properties		Wound Healing		Ref.
Polysaccharides	Component	Bioactive Agent	Concentration	Mechanism	Time (Day)	
SA	PF127 CS	Curcumin	5 mg mL^−1^	Antibacterial Antioxidant	20	[95]
HA	HP407; FND; EGFN; PHNB	-	-	Anti-inflammatory Pro-angiogenesis Antioxidant Antibacterial	15	[100]
BCL	-	*-*	-	Antibacterial	21	[105]
CA		Dimethyloxallyl Glycine Silver nanoparticles	2.5% (*w*/*w*) 3.2% (*w*/*w*)	Pro-angiogenesis Antibacterial	-	[107]

CA = cellulose acetate; BCL = bacterial cellulose; EGFN = epidermal growth factor loaded-nanoparticles; FND = fluorocarbon nanodroplets; HA = hyaluronic acid; HP407 = hexamethylene diisocyanate-Poloxamer 407 copolymer; PHNB = polyhexamethylene biguanide; SA = sodium alginate.

### 3.3. Mixing of Polysaccharides-Based Hydrogel in the Treatment of Diabetic Wounds

Combining two or more bioactive polymers together in a single tridimensional structure represents an innovative strategy benefitting from their different mechanisms of actions in wound repair, thus maximizing the potential for proper wound healing (Table 7).

In this regards, ionic interactions between the oppositely charged aqueous solutions of CH and SA biopolymers can be useful when employed in the preparation of polyelectrolyte complexes, without the need of reaction initiators or catalysts [108]. In vivo experiments performed on diabetic mice demonstrated their ability to improve the healing rate and skin quality and better results were obtained at higher CH concentration, able to ensure a complete wound recovery after 14 days. CH was also successfully mixed with carboxymethyl cellulose (CMC) at different concentrations (0.3–0.9% *w*/*w*) to synthesize a tridimensional hydrophilic scaffold proposed as a mequinol carrier in the treatment of diabetic wounds [109]. Mequinol is an active phenolic molecule known as a potent antioxidant able to prevent lipid peroxidation and protect cells against oxidative stress in diabetic wounds [110]. The inhibitory activity of multifunctional hydrogel on the growth of model bacteria (*S. aureus* and *E. coli*) was dose-dependent and increased at higher concentrations, while the loading of drug molecules significantly improves the antioxidant (investigated as scavenger activity against DPPH radical) and anti-inflammatory features.

K-carrageenans (KCA), a family of biocompatible, non-toxic, and biodegradable linear sulfated polysaccharides extracted from red edible seaweeds, are largely employed in combination with other biopolymers to form biomaterial for several pharmaceutical and biomedical purposes [111]. Self-crosslinked CH/KCA/PVA-based biomimetic membrane dressings loaded with cefotaxime sodium (active against Gram-positive, Gram-negative bacteria, and some strains of anaerobic bacteria responsible for bone and soft tissue infections) were synthesized for potential diabetic burn wound healing [112]. The drug loaded hydrogel exhibited a retained release for up to 24 h, providing protection against bacterial proliferation (*P. aeruginosa*, *E. coli*, and *S. aureus*). In vivo experiments performed on a diabetic rat burn model displayed rapid (98% after 14 days) wound healing with increased subcutaneous granulating tissue richness.

**Figure 5:**
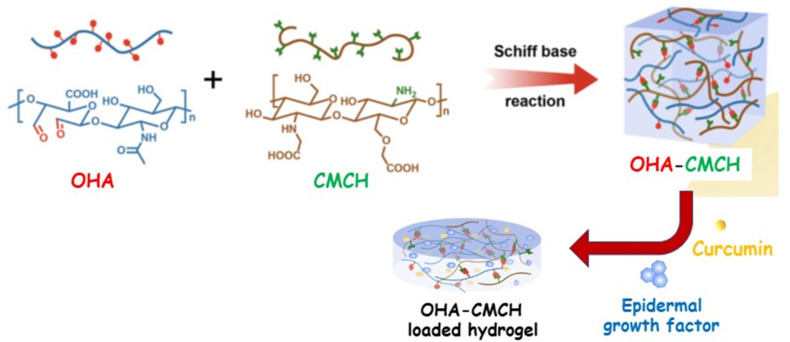
Schematic representations of the oxidized hyaluronic acid-carboxymethyl chitosan (OHA-CMCH) curcumin and epidermal growth factor loaded hydrogel fabricated via the reversible Schiff base reaction between OHA and CMCH [113].

A multifunctional hydrogel based on HA and CMCH linked by Schiff base to the oxidized HA (OHA) was proposed as a transport device to locally co-deliver curcumin and epidermal growth factor (Figure 5) [113].

Curcumin was constantly released to lighten inflammation process and oxidative stress in the initial stage of wound healing, while a sustained release of epidermal growth factor maintained late proliferation and extra cellular membrane remodeling. Additionally, OHA and CMCH displayed outstanding intrinsic antibacterial and hemostatic properties. A similar approach was investigated by Ou and co-workers, who designed a novel hydrogel dressing possessing multiple features, including injectability, self-healing, conductivity, and antibacterial properties to treat infected diabetic wounds [114]. Specifically, a hydrogel network was synthesized by the dynamic Schiff base reaction of the amino group of N-carboxyethyl chitosan (CECH), the aldehyde group of oxidized hyaluronic acid. In addition, the employment of graphene oxide improved the mechanical properties of the hydrogel, also conferring exceptional conductivity and immune regulation to the tridimensional system [115]. This device was loaded with Polymyxin B, which increases the antibacterial performances by synergistic action with the CECH moieties, to address complex chronic wounds, such as infected diabetic wounds.

To synthesize the hydrogel dressing with enhanced antibacterial activity, high performing QCH was successfully combined with OHA, while α-lipoic acid loaded cyclodextrins were introduced to couple both antimicrobial and antioxidant capacities [116]. QCH-OHA-α-lipoic acid hydrogel promptly promoted diabetic wound healing in vivo, confirming that wound edges were significantly narrowed at day 11 post-wounding. More recently, tridimensional system based on QCH and oxidized β-glucan (OGL) were loaded with Polydomaine nanoparticles [117] and Poly(tannic acid) nanorods [84]. Both designed hydrogels involved various therapeutic mechanisms (pro-angiogenesis, antioxidant, antibacterial) into a single dressing, to optimize the effectiveness of the treatment and providing an effective and simple therapy plan for healing diabetic infected wounds into a limited time.

**Figure 6:**
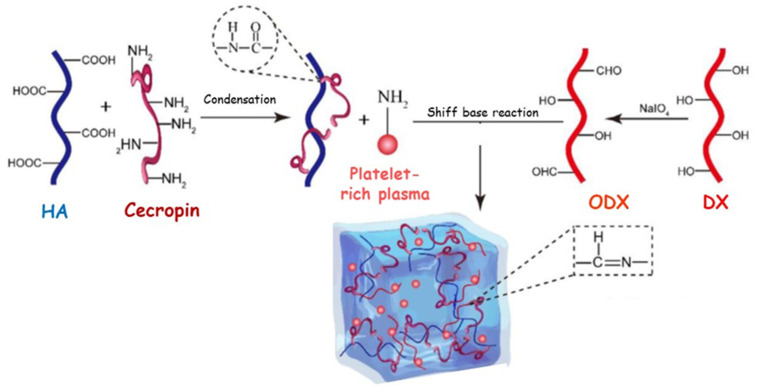
Schematic illustration of the synthesis of Hyaluronic acid (HA)-Cecropin and Oxidized dextran (ODX) and their crosslinking reaction to fabricate ODX/HA-Cecropin/Platelet rich plasma hydrogel [119].

Alternatively, pH-responsive and biocompatible calcium alginate (CaAL) hydrogel loaded with protamine nanoparticles and OHA was effectively synthesized [118]. A considerable reduction of bacteria-induced chronic inflammation at the wound site, as well as a consistent promotion of the wound-healing (more than 90% after 14 days) was observed. Specifically, the enclosing of the antibacterial protamine nanoparticles displayed a remarkable inhibitory effect against *S. aureus* and *E. coli* bacteria, while OHA significantly improved the migration of the endothelial cells and the secretion of vascular endothelial growth factors. More recently, antimicrobial peptides (Cecropin) and platelet-rich plasma were co-delivered from a tridimensional network based on oxidized dextran (ODX) and HA opportunely modified with the antibacterial arm (Figure 6) [119]. In vitro experiments clearly highlighted the ability of the device to inhibit pathogenic bacterial strains (*P. aeruginosa*, *E. coli*, and *S. aureus*) by controlling the release rate of both growth factors and antibacterial species. Furthermore, a significant regulation of the inflammation process, as well as an accelerated collagen deposition and an improvement in the angiogenesis process were recorded in in vivo analyses performed on diabetic mouse infections.

Finally, Ag-coated ZnO-loaded hydrogel dressings were prepared by involving KCA, konjac glucomannan (KG), and GO as reinforcements to improve the mechanical resistance of the tridimensional network [120]. Antibacterial in vitro evaluation against *E. coli* and *S. aureus* microbial species displayed a reduction equal to 96% for Gram-positive bacteria cells, while cell death was more than 98% for Gram-negative bacterial cells.

**Table 7:** Antibacterial polysaccharides-mixed hydrogels employed in the diabetic wound healing.

Hydrogel Composition			Delivery Properties		Wound Healing		Ref.
Polysaccharides		Others	Bioactive Agent	Concentration	Mechanism	Time (Day)	
Component_1_	Component_2_						
CH	AL	-	-	-	Antimicrobial	14	[108]
CH	CMC	-	Mequinol	0.3% (*w*/*w*)	Antioxidant Antibacterial	-	[109]
CH	KCA	PVA	Cefotaxime sodium	1% (*w*/*w*)	Antibacterial	21	[112]
CMCH	OHA	-	Modified curcumin Epidermal growth factor	-	Anti-inflammatory Pro-angiogenesis Antioxidant Antibacterial	15	[113]
CECH	OHA	GO	Polymyxin B	1% (*w*/*w*)	Antioxidant Antibacterial	18	[114]
QCH	OHA	-	α-lipoic acid	1–5% (*w*/*w*)	Antioxidant Antibacterial	11	[116]
QCH	OGL	-	Polydomaine nanoparticles	0.5–2 mg mL^−1^	Pro-angiogenesis Antioxidant Antibacterial	15	[117]
QCH	OGL	-	Poly(tannic acid) nanorods	0.5–2 mg mL^−1^	Pro-angiogenesis Antioxidant Antibacterial	35	[84]
CaAL	HAO	-	Protamine	2 mg mL^−1^	Pro-angiogenesis Antibacterial	14	[118]
HA-peptide modified	ODX	-	Platelet-rich plasma	14% (*v*/*v*)	Pro-angiogenesis Antibacterial	14	[119]
CMC	KCA KG	GO	Ag-ZnO nanoparticles	2.0% (*w*/*w*)	Antibacterial	-	[120]

AL = alginate; CaAL = calcium alginate; CH = chitosan; CECH = carboxyethyl chitosan; CMC = carboxymethyl cellulose; CMCH = carboxymethyl chitosan; GO = graphene oxide; KCA = κ-carrageenan; KG = konjac glucomannan; HA = hyaluronic acid; HAO = hyaluronan oligosaccharides; ODX = oxidized dextran; OGL = oxidized β-glucan; OHA = oxidized hyaluronic acid; QCH = quaternized chitosan; PVA = polyvinyl alcohol.

### 3.4. Gelatin-Based Hydrogels in the Treatment of Diabetic Wounds

Gelatin (GL) represents a raw material that is useful when employed in the preparation of hydrogel dressings for diabetic wounds. GL is a biocompatible and easily enzymatically degraded natural protein extracted from collagen that is largely used as a biomimetic peptide able to promote cell adhesion, differentiation, and proliferation [91]. In addition, chemical functionalities on the polypeptide backbone can be exploited to efficiently preparate GL-based hydrogel dressings with tailored features [121]. GL-based hydrogels proposed in the treatment of diabetic wounds are listed in Table 8.

GL was successfully crosslinked with polyacrylamide for the preparation of an extensive temperature-tolerant, water retained, and adhesive hydrogel dressing inserted with antibacterial ε-polylysine chains [122]. The dressing displayed great mechanical properties, exceptional adhesiveness in a wide temperature range (from −20 to 60 °C), and excellent antibacterial features against *E. coli* and *S. aureus*. In particular, experiments performed on diabetic rats highlighted an accelerated wound healing by supporting collagen deposition and angiogenesis, as well as avoiding bacterial infection due to the presence of the ε-polylysine chains.

To involve gelatin chains in a radical crosslinking process, suitable polymerizable functional groups were inserted in the polypeptide backbone. In particular, self-healing, antibacterial, and adhesive dressings were prepared by involving gelatin methacrylate (GL-MA), adenine acrylate, and CuCl_2_ in a covalent/coordination crosslinking process [123]. Specifically, acrylic groups generate the covalent network, while Cu^2+^ and carboxyl groups stabilize the structure with hydrogen bonds. The dressing displayed remarkable antibacterial properties against *E. coli* and *S. aureus* bacteria, and the efficient hemostatic performance significantly stimulated the healing processes in a full-thickness skin diabetic wound model. A multifunctional hydrogel was prepared by starting from photoinduced radical crosslinking of GL-MA and is proposed as a carrier of cerium-containing bioactive glass [124]. Bioactive glass has been extensively applied in tissue engineering, due to its capacity to stimulate angiogenesis and repair soft tissue wounds [125], while cerium displayed significant antibacterial properties against both Gram positive and Gram negative bacteria [126]. Alternatively, in situ UV-initiated hydrogel was synthetized by chemical crosslinking of GL-MA and zwitterionic hyperbranched terpolymer prepared by a dynamically controllable reversible addition-fragmentation chain transfer [127]. This carrier was successfully proposed to transport Ag^+^ ions complexed with the anti-inflammatory ascorbyl palmitate nanosheets. The subsequent in situ reduction of silver ions to Ag nanoparticles provided a system able to preserve wounds from the antibacterial infections. Antibacterial and anti-inflammatory actions, as well as angiogenesis promotion in the treatment of diabetic wounds, were evaluated by employing a pH and reactive oxygen species responsive hydrogel synthesized by grafting 3-carboxy-phenylboronic acid to the GL chain and subsequent crosslinking of the conjugate with poly(vinyl alcohol) [128]. Vancomycin-conjugated silver nanoclusters and pH-sensitive micelles loaded with nimesulide were efficiently incapsulated into the tridimensional network to prepare a multifunctional hydrogel, showing remarkable antibacterial properties against *S. aureus* and *P. aeruginosa*. In vivo experiments performed on diabetic rats returned a significant acceleration of the wounds due to hemostatic and anti-inflammatory action of the loaded therapeutics, while the antibacterial features avoid the occurrence of infections.

To synthesize a hydrogel dressing with antioxidant properties, specific functional moieties were grafted into the GL backbone. In particular, chemical functionalization with 2,3,4-trihydroxybenzaldehyde and simultaneous ionic crosslinking by iron (III) ions were carried out on a tridimensional network with both antioxidant and antibacterial properties [129]. Similarly, a multifunctional hydrogel with significant scavenging properties and photothermal antibacterial capacity based on oxidized dextran, gallic acid-grafted gelatin, and Fe^3+^ ions, was proposed for the treatment of infected wound in diabetic mice [130]. In this way a double-crosslinked network was synthesized by the dynamical Schiff-base bonds involving the aldehyde groups in the dextran and amino groups of the functionalized GL, while metal coordination bonds were formed between Fe^3+^ ions and hydroxyl carboxyl groups of the protein. The in vivo experiments enhanced the complete re-epithelialization of *S. aureus*-infected wound in diabetic mice in 18 days by eliminating the infection, alleviating oxidative stress and inflammation, and accelerating angiogenesis.

**Table 8:** Antibacterial gelatin-based gelatin hydrogels employed in the diabetic wound healing.

Hydrogel Composition		Delivery Properties		Wound Healing		Ref.
Protein	Component	Bioactive Agent	Concentration	Mechanism	Time (Day)	
GL	PAC	PLy	10% (*w*/*v*)	Antibacterial	18	[122]
GL-MA	AA	Cu^2+^	0.5–1.5 mg mL^−1^	Antibacterial	21	[123]
GL-MA	-	Ce-BG	1% (*w*/*v*)	Antibacterial Pro-angiogenesis	21	[124]
GL-MA	PEGDA; VI; DMAPS; AP	Ag nanoparticles	10% (*w*/*w*)	Antibacterial Anti-inflammatory	14	[127]
GL-CPBA	PVA	VAN-AgNCL NIM	0.04 mg mL^−1^ 0.03 mg mL^−1^	Pro-angiogenesis Antibacterial Anti-inflammatory	14	[128]
GL-THB	Fe^3+^	-	-	Antibacterial Antioxidant Pro-angiogenesis	21	[129]
GL-GA	ODX	Fe^3+^	0–14 mM	Pro-angiogenesis Antibacterial Antioxidant	18	[130]

AA = adenine acrylate; AP = ascorbyl palmitate; Ce-BG = cerium-containing bioactive glass; DMAPS = 2-(N-3-sulfopropyl-N,N-dimethyl ammonium) ethyl methacrylate; GL = gelatin; GL-CPBA = gelatin- 3-carboxy-phenylboronic acid; GL-GA = gelatin-gallic acid; GL-THB = 2,3,4-trihydroxybenzaldehyde-tethered gelatin; GL-MA = Gelatin methacrylate; NIM = nimesulide; ODX = oxidized dextran; PAC = polyacrylamide; PLy = ε-polylysine; PEGDA = poly(ethylene glycol) diacrylate; PVA = poly(vinyl alcohol); VAN-AgNCL = vancomycin-conjugated silver nanoclusters; VI = 1-vinylimidazole.

## 4. Conclusions and Future Perspective

This review summarized the main natural compounds and biopolymers employed in wound healing, which had interesting antibacterial activity against the most common bacterial species, both gram positive and negative. A strong antibacterial property helps the natural wound healing process, mainly when it is delayed due to pathological conditions, such as Type 2 Diabetes Mellitus. Natural compounds are useful tools in this context, and their low bioavailability is easily overcome by inclusion in biopolymers. Moreover, several biopolymers show antibacterial and wound healing activity, and have become precious remedies to reach wound closure. In general, the different bioactive compounds transported by the reviewed natural biomaterials should represent a good starting point for the development of novel broad-spectrum devices for wound healing and management. Hydrogel dressings are supposed to be extensively employed in diabetic wound treatment. However, innovative studies into wound healing should be performed by combining aspects from the complementary areas of the material science, cellular and molecular biology, as well as electrical bioengineering. In order to ensure further improvement in the available tools for diabetic wound healing, novel biomacromolecules resembling the extra-cellular membrane should be considered. The preparation of multifunctional hydrogels able to combine an intelligent response to the microenvironment of the wound with specific electronic platforms will be able to implement real-time monitoring of the wounds. In this way, peculiar aspects such as different diabetic patients, degree of the wound, and healing capacity of wounds will be taking into account in the customized design of the hydrogel dressings.

## Figures and Tables

**Table 1:** Phenolic compounds.

Poliphenol	Structure	Characteristics
Quercetin	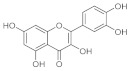	It modulates the activity of fibroblasts. It up-regulates vascular the endothelial growth factor and transforms growth factor-β1. It is a wound healing agent for diabetic scars. Limitations: low bioavaibility and low systemic and topic absorption
Curcumin	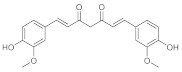	It is involved in tissue remodeling, the formation of granulation tissue, and collagen deposition. It can induce the regeneration of epithelial tissue and increases fibroblasts proliferation and vascular density. It is poorly absorbed following oral administration. It is involved in extensive first-pass metabolism. It is a light-sensitive molecule. Topical formulations are preferred.
Pinocembrin	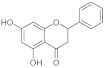	It is able to modulate the production of inflammatory cytokines. It can accelerate in vitro skin wound healing, improving the migration of keratinocytes. Limitations are associated to its low bioavavibility.
Chrysin	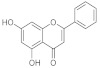	It can take a reduction of p53 and iNOS expression. Limitations: low stability in vivo due to its poor acqueos solubility and low bioavaibility Assocations with polymers or other compounds can ameliorate the situation.
Luteolin	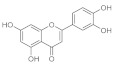	It can inhibit TNF-α and IL-6 and iNOS secretion in LPS-activated macrophages. It can modulate IGF, PDGF, and FGF. It can also suppress NETs in activated human neutrophils, and improve immune system by inhibiting the production of ROS. Limitations: it has low bioavability so local use is preferred but also other ways can be undertaken, such as intraperitoneal injection for systemic effects.
Catechin and Epigallocatechin-3-gallate	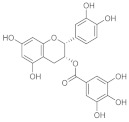	They are able to reduce TNF-α secretiona and NFκB activity. They can inhibit the production of NO regulating inflammatory processes. Limitations: they have poor systemic absorption, bad biodistribution, suffer of first-pass metabolism, and have low stability, which take to the formation of degradation products. Nanoformulation can get around these problems.
Tannic acid	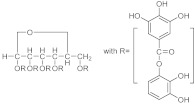	It can inhibit lipid oxidation by removing free radicals. Its application can be topical, local, or systemic. It can be combined with polymers to become more resistant to proteolytic enzymes. There are controversial hypotesis about its cytotoxic effects but at the current state it is considered a safe food additive.
Terpinolene and α-phellandrene	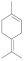 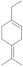	They can improve the migration and proliferation of fibroblasts, suppress IL-6 and TNF-α, inhibit NO production, and suppress NF-κB activity. Limitations: They are lipophilic so they present low bioavaibility but nanoformulation are used to improve their use.
Thymol	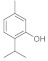	It improves the edema formation and the influx of leukocytes to the wound area. It improves granulation reaction. Limitations: it is rapidly absorbed in vivo, but nanoformulations can be used to increase solubility and stability.
Taspine	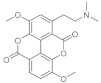	It shows chemotactic properties on fibroblasts. Limitations: Low bioavaibility, but the association with other compounds or polymers can be used in its advantage in the use of the molecule.

**Table 2:** The effect of quercetin glycoside on excision wound [% wound closure].

Day	Group 1	Group 2	Group 3
0	0%	0%	0%
4	24.30%	37.50%	33.33%
8	29.00%	58.33%	65.07%
12	66.66%	79.85%	87.50%
16	83.33%	91.66%	95.83%

**Table 3:** Antibacterial screening using the agar disc diffusion method.

Curcumin + Chitosan-PVA Mixture	*E. coli* ^a^	*P. itocida* ^a^	*B. subtilis* ^a^	*S. aureus* ^a^
Curcumin (10 mg/mL)	12 ± 2.45	14 ± 3.5	11 ± 1.23	13 ± 0.95
Curcumin (20 mg/mL)	15 ± 4.23	16 ± 2.52	14 ± 2.18	14 ± 0.90
Curcumin (30 mg/mL)	17 ± 5.50	20 ± 2.24	13 ± 3.27	15 ± 2.50
Curcumin (10 mg + Chitosan-PVP 80)	22 ± 3.56	24 ± 0.90	15 ± 3.52	17 ± 0.96
Curcumin (20 mg + Chitosan-PVP 80)	25 ± 1.90	23 ± 0.8	17 ± 0.54	20 ± 2.50
Curcumin (30 mg + Chitosan-PVP 80)	28 ± 2.7	26 ± 3.8	25 ± 3.1	23 ± 1.50
Chitosan-PVA 80	18 ± 0.5	20 ± 0.7	13 ± 0.4	18 ± 3.59
Rifampicin	36 ± 0.9	32 ± 1.4	30 ± 2.8	36 ± 4.3

^a^ Bacterial strains: *E. coli* = *Escherichia coli*; *P. itocida* = *Pasturellamu itocida*; *B. subitilis* = *Bacillus subtilis*; *S. aureus* = *Staphylococcus aureus* (the values are the mean of triplicate samples (n = 3) ± S.D.

**Table 4:** Main characteristic of the most important polymeric compounds employed in diabetic wound treatment.

Polymer	Structure	Characteristics
Chitosan	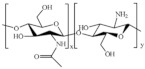	Antimicrobial, wound healing, antidiabetic, biodegradability, nontoxicity, biocompatibility, anti-inflammatory, hemostasis
Alginate	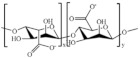	Antimicrobial, moisture absorbing, hydrophilicity, biocompatibility, gelation
Hyaluronic acid	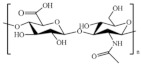	Antimicrobial, biodegradability, anti-adhesive, viscoelasticity lubricity, biocompatibility, immunostimulatory
β-Glucan	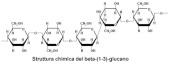	Antiproliferative, blood glucose regulation, immunomodulatory
Cellulose	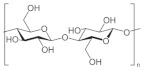	Biodegradable, biocompatible, non-carcinogenic, non-toxic, retain moisture, absorb exudates, gelation
Konjac glucomannan	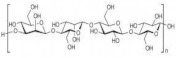	Biocompatibility, gelling agent, biodegradability
